# Assessment of patient information guides generated by LLMs for common cardiological procedures

**DOI:** 10.21542/gcsp.2025.26

**Published:** 2025-05-15

**Authors:** Suppraja Soundarrajan, Karine Vartanian, Rahul Bhakle, Thanuja Katakam, Kinnera Dhanwada, Karansher Singh Randhawa, Nikhitha Puvvala

**Affiliations:** 1 IGovernment Medical College, Omandurar Govt. Estate, Chennai 600002, India; 2 Southern California Hospital Heart Institute, 3831 Hughes Ave, Suite 105, Culver City, CA, USA; 3 Medical College Baroda, SSG Hospital, Vadodara, Gujarat, India; 4 SRM Medical College Hospital and Research Center, Chennai, India; 5 Mysore Medical College and Research Institute, Mysore, Karnataka, India; 6 Government Medical College, Patiala, India; 7 Malla Reddy Institute of Medical Sciences, Hyderabad, Telangana, India

## Abstract

**Introduction**: The use of artificial intelligence (AI) has advanced rapidly in the field of cardiology owing to its ability to process complex data and analyze electrocardiograms, echocardiography, and cardiac testing. AI tools, such as ChatGPT and Google Gemini, can provide evidence-based treatment recommendations using concise language, which can help in the early diagnosis of disease.

**Methodology:** In this cross-sectional study, patient information brochures for three cardiological procedures (ECG, 2D echocardiography, and exercise stress testing) were generated using ChatGPT and Google Gemini. The total word count, sentence count, average words per sentence, and syllables for words were assessed using the Flesch-Kincaid Calculator. The similarity of the text was determined using the Quill Bot plagiarism tool. The reliability of the generated responses was analyzed and graded using the Modified DISCERN Score, which is a 5-point rating system that uses a set of uniform standards to assess the accuracy and dependability of consumer health-related data. Statistical analysis was performed using RStudio v4.3.2. Additionally, the simplicity and reliability scores were compared using Pearson’s Coefficient of Correlation. The unpaired *t*-test was used to compare the responses.

**Results:** Responses generated by ChatGPT and Google Gemini were observed to have no significant difference in the word count (*P* = 0.59), sentence count (*P* = 0.74), average word per sentence (*P* = 0.79), grade level (*P* = 0.06), similarity (*P* = 0.45), and reliability scores (*P* = 0.38) between ChatGPT and Google Gemini. However, the ease score was significantly better for Google Gemini-generated responses than for ChatGPT (*P* = 0.0044), indicating that the responses generated by Google Gemini are more easily readable and understandable.

**Conclusions:** The study found a statistically significant difference between the average syllables per word and ease score. No significant differences were observed in the number of words, sentences, average words per sentence, grade level, similarity, or reliability scores. More AI technologies need to be evaluated in future studies, which should cover a wider range of illnesses.

## Introduction

Cardiac diagnostics employs a range of non-invasive tests to comprehensively assess heart health and detect potential issues. Among these, an electrocardiogram (ECG) records the heart’s electrical activity with electrode patches on the wrists, ankles, and six chest points, providing 12 different perspectives to detect irregular rhythms, stress, or damage to the heart^1^. The process of ECHO is to utilize sound waves to generate images of the heart, which are then recorded on a film to identify any structural irregularities^2^. Stress testing is essential for detecting and categorizing the risk of coronary artery disease (CAD). It helps determine the need for coronary angiograms by considering specific patient characteristics and deciding between standard exercise testing and stress imaging^2^. Screening tests are valuable for promptly detecting cardiovascular disorders, enabling timely intervention and targeted treatment. This ultimately reduces the occurrence of cardiovascular events in people who are at risk^3^.

ChatGPT, developed by OpenAI, is a language model that employs deep learning methods to generate human-like responses by processing extensive amounts of human-language data^4^. Gemini, built by Google, is a versatile AI system that can handle several types of inputs, including audio, video, and image data, in addition to text-based jobs. This makes it highly suitable for a wide range of tasks^5^. Artificial intelligence (AI) simplifies intricate problems in a convenient and user-friendly manner, making it appropriate for frequent utilization from any location^6^. However, its integration into healthcare requires careful consideration of limitations, such as increased dependency, bias, privacy concerns, and trust issues. Continuous parameter fine-tuning may compromise transparency, potentially impacting clinical diagnosis and reducing critical interactions between healthcare professionals and patients, which is essential for maintaining quality care and trust in effective healthcare delivery^7^.

A patient information guide is needed to educate subjects regarding non-invasive cardiac tests, such as ECG, ECHO, and stress testing, which helps identify and exclude heart conditions and determine the need for medications for prevention and treatment^8^. AI educates individuals through multimedia education delivery in the form of videos, voice, and print, tailored to different literacy levels and languages, enhancing understanding, and promoting shared decision-making to improve health conditions, while considering and monitoring factors such as patient emotional state and information accuracy^9^.

### Aims and objectives

To compare the ChatGPT- and Google Gemini-generated patient education guides on exercise stress tests, transthoracic echocardiography, and electrocardiograms based on readability and ease of understanding.

## Methodology

A cross-sectional study was conducted from June 12 to June 19, 2024, to compare ChatGPT and Google Gemini-generated responses for writing a patient education guide on the commonly utilized diagnostic modalities in a cardiology service. This study utilized AI software without any involvement of human subjects or use of personalized information, thus exempting the study from the need for ethics committee approval.

Three commonly utilized diagnostic modalities in cardiology, namely, the exercise stress test, transthoracic echocardiography (ECHO), and electrocardiogram (ECG), were selected for this study. Two AI tools, ChatGPT version 3.5, and Google Gemini, were utilized for the generation of brochures for patient education^10,11^. Both large language models (LLMs) were given a similar, specific prompt. The prompt was—‘Write a patient education guide for Exercise stress test.’ Similar prompts were used for ECHO and ECG. The generated responses were tabulated in Microsoft Word.

These responses were then assessed and graded for word count, ease of understanding, and readability of the information generated using the Flesch-Kincaid calculator^12^. Additionally, the Quill Bot plagiarism tool was used to calculate the similarity of the text generated by these Large Language Models (LLM) to the text written in published articles on the Internet^13^. Finally, the Modified DISCERN Score was used to analyze and grade the reliability of the responses generated. The Modified DISCERN Score is a 5-point scoring tool consisting of a set of standardized criteria to evaluate the reliability and quality of consumer health-related information available on online platforms. It scores literature based on relevance, accuracy, clarity, biases, date of publication, and the use of standardized references^14^.

The data were exported to a Microsoft Excel spreadsheet after being compiled in a tabulated form in Microsoft Word. Subsequently, the R core team (2023) conducted statistical analysis of the data using R version 4.3.2. Vienna, Austria: R: A Language and Environment for Statistical Computing. An unpaired *t*-test was employed to compare the responses generated by ChatGPT and Google Gemini using this software. Statistical significance was set at *p* < 0.05. Pearson’s Coefficient of Correlation was also used to compare simplicity and reliability scores. Appropriate pivot charts and graphical charts were generated to illustrate the results of this study in relation to the evaluated parameters.

## Results

Table 1 presents the characteristics of the responses generated by ChatGPT and Google Gemini. There was no significant difference in the word count (*P* = 0.59), sentence count (*P* = 0.74), average word per sentence (*P* = 0.79), grade level (*P* = 0.06), similarity (*P* = 0.45) and reliability scores (*P* = 0.38) between ChatGPT and Google Gemini. However, the ease score was significantly better for Google Gemini-generated responses than for ChatGPT (*P* = 0.0044), indicating that the responses generated by Google Gemini are easily readable and understandable.

**Table 1:** Characteristics of responses generated by ChatGPT and Google Gemini.

Variables	ChatGPT		Google Gemini		*P* value^*^
	Mean	Standard Deviation	Mean	Standard Deviation	
Words	447.00	67.51	420.70	38.08	0.5957
Sentences	41.00	21.63	36.33	5.51	0.7484
Average Words per Sentence	12.40	4.29	11.67	1.10	0.7984
Average Syllables per Word	1.80	0.00	1.57	0.06	0.0198^*^
Grade Level	10.50	1.63	7.40	0.56	0.0691
Ease Score	41.97	4.33	62.43	4.31	0.0044^*^
Similarity %	49.57	10.61	40.23	16.17	0.4571
Reliability Score	2.67	0.58	2.0	1.00	0.3868

**Notes.**

* *t*-test. *P*-values < 0.05 are considered statistically significant.

**Figure 1.**
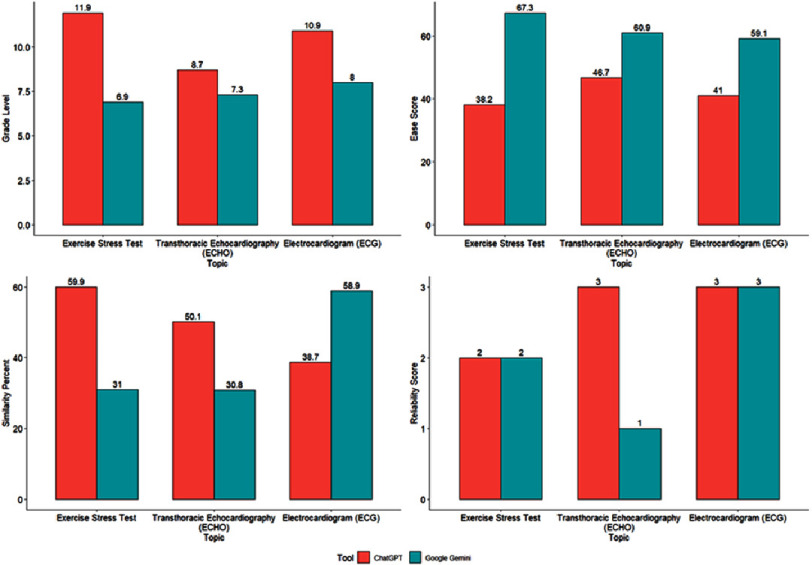
Comparisons between grade level, ease score, similarity percent, and reliability score for the patient education guide produced by ChatGPT and Google Gemini.

Figure 1 shows a graphical representation of the comparison between the grade level, ease score, similarity percentage, and reliability score for the patient education guide generated by ChatGPT and Google Gemini. In grade level, ChatGPT exhibited superior performance over Google Gemini in all cardiological investigational modalities. Conversely, the ease score was significantly better for Google Gemini. There was no considerable difference in the reliability of the exercise stress test and electrocardiogram; however, for Transthoracic Echocardiography, the ChatGPT showed a significantly higher score than Google Gemini. Additionally, the ChatGPT similarity percentage was higher in all investigational modalities, except for electrocardiography.

## Discussion

AI chatbots are unquestionably useful tools for the future of healthcare, improving productivity, and expanding healthcare personnel skills. They may help to provide patient education and illness management assistance^15^. This increases the need for medical professionals to assess the appropriateness and report results, which should improve the quality of the data generated. Patient education brochures that are brief and concise, while providing all high-yield information, are deemed optimal. The investigation was conducted by assessing the total word count and the average word count per sentence. The study found that ChatGPT had somewhat higher values (447 words, 12.40 average) compared to Google Gemini (420.70 words, 11.67 average). In contrast to other similar studies, ChatGPT consistently exhibited a reduced mean word count compared with Gemini. The outcome may be the result of a distinct field of research^16,17^.

The Flesch Reading Ease score was calculated using the following equation: 206.835–1.015 (total words/total sentences) − 84.6 (total syllables/total words). Policy writers, research communicators, and digital marketers use these scores to determine the level of ease with which a target audience will comprehend a given piece of information^12^. The current study demonstrates a statistically significant disparity in the ease ratings of the two AI tools, with Google Gemini achieving a higher score of 62.93, which corresponds to a level of education typically attained in the eighth and ninth grades. Furthermore, yet another cross-sectional investigation revealed that ChatGPT obtained the lowest score, hence confirming the difficult nature of ChatGPT^18^.

Integrating generative AI into patient education is not without its difficulties, including ethical concerns, copyright concerns, and plagiarism concerns^19^. Consequently, the similarity score for each response provided by the two AI tools was assessed. According to the findings, ChatGPT achieved a superior score of 49.57, while Google Gemini scored 40.23. A separate study also noted a comparable pattern, where ChatGPT achieved a similarity score of 34%, indicating significant problems with plagiarism^20^.

AI can potentially expand the clinical implications, which include estimation of bone age on radiographic exams, diagnosing treatable retinal diseases on optical coherence tomography, or quantifying vessel stenosis and other metrics on cardiac imaging^21^. Healthcare professionals may be able to handle more difficult activities by automating labor- and time-intensive but conceptually simple jobs, which would be an improved use of human resources by the addition of AI to generate patient education materials and other expanded uses in the future.

This study employed the DISCERN score as a means of evaluating the pertinence and dependability of the responses produced by the two AI systems^22^. According to this study, ChatGPT outperformed Google Gemini in terms of Transthoracic Echocardiography scores. The average value of ChatGPT was marginally higher (2.67) compared to Google Gemini, with a *p*-value of 0.3868, indicating that the difference between them is not statistically significant. In another cross-sectional investigation, it was observed that ChatGPT had superior performance in terms of reliability compared to the other models. This was demonstrated by a higher DISCERN score^23^.

Although ChatGPT-generated education is currently less accessible than provider-written content, patients will likely reach out for them more frequently in the future. Some AI prompts can simplify learning materials to meet national standards, while accommodating individual literacy, according to a study^24^.

## Limitations

The study’s main limitation is that it only examined two AI tools. The quality and neutrality of the patient guides generated may be influenced by the technical limitations of both AI models, such as potential biases in training data. It is necessary to assess additional AI-Large Language Models (LLM). Additionally, the scope of this study is restricted to the examination of patient manuals for three distinct cardiological procedures: ECG, ECHO, and stress testing. The findings may not be applicable to other medical procedures or specialties, and future research could concentrate on other diseases, interventions, and/or procedures. Medical professionals have not reviewed the guides generated in this study, which is essential for guaranteeing the quality of patient education. Finally, ChatGPT is an illustration of an artificial intelligence technology that may not offer the most recent content. Given the constant evolution of the medical field, AI technologies necessitate frequent updates to medical information in order to maintain their credibility as a reliable source of information.

## Conclusion

This study demonstrates that the average syllables per word and comfort score of the patient education guides generated by ChatGPT and Google Gemini on Exercise Stress Tests, Transthoracic Echocardiography, and electrocardiograms are statistically significantly different. The responses generated by the two AI tools do not exhibit any substantial differences in terms of the number of words, sentences, average words per sentence, grade level, similarity, and reliability scores.

Future research must evaluate an even greater number of AI tools. In addition, they must encompass a broader spectrum of diseases and frequently employ diagnostic methods. Verification of the sources utilized by AI tools for the information they provide is imperative. Whether these tools can accurately provide information in accordance with the most recent guidelines to prevent misinformation and whether this information is comprehensible to the general public, who may not be able to understand complex medical terms, must be evaluated.
